# Prevalence and associated factors of uncontrolled hypertension among hypertensive patients: a nation-wide survey in Thailand

**DOI:** 10.1186/s13104-019-4417-7

**Published:** 2019-07-04

**Authors:** Boonsub Sakboonyarat, Ram Rangsin, Anupong Kantiwong, Mathirut Mungthin

**Affiliations:** 1 Department of Military and Community Medicine, Phramongkutklao College of Medicine, Bangkok, 10400 Thailand; 2 Department of Pharmacology, Phramongkutklao College of Medicine, Bangkok, 10400 Thailand

**Keywords:** Uncontrolled hypertension, Prevalence, Associated factors, Nationwide, Thailand

## Abstract

**Objectives:**

The objectives of the research were to determine the prevalence and factors associated of uncontrolled blood pressure among Thai hypertensive patients in a nationwide survey.

**Results:**

A total of 65,667 patients with hypertension were included in this study. The greater proportion of participants, 40,834 (62.2%), were females. The average age of participants was 63.9 ± 11.1 years. Uncontrolled hypertension was detected among 16,122 patients (24.6%; 95% CI 24.2–24.9). Among males and females, uncontrolled hypertension was 25.6% (95% CI 25.1–26.2) and 23.9% (95% CI 23.5–24.3) respectively. Multivariate analysis showed that the uncontrolled hypertension was significantly associated with being male, age, regions, hospital levels, diabetes comorbidity, higher body mass index, low density lipoprotein cholesterol level and the number of antihypertensive medications.

## Introduction

Hypertension is one of the most common cardiovascular disorders worldwide. Globally, 30.8% of adults have hypertension, and the estimated prevalence of hypertension among males and females is 32.1% and 29.5%, respectively [1]. Hypertension is a major modifiable risk factor for heart diseases, stroke, end stage renal failure and peripheral vascular disease. The Thai National Health Examination Survey (NHES) 2004 and the NHES 2009 data indicated that it affects nearly one in every five Thai adults [2]. Information on controlling blood pressure (BP) is available worldwide, and the prevalence of controlled BP reported in the US, UK, China and Japan were 68.9%, 60.8%, 37.5% and 37.1%, respectively [1, 3]. However, limited information is available regarding the prevalence of uncontrolled hypertension in Thailand especially in nationwide studies. We aimed to use the information from the Thailand Diabetes Mellitus/Hypertension (DM/HT) of the Medical Research Network of the Consortium of Thai Medical Schools (MedResNet) to determine BP control and risk factors associated with uncontrolled BP among Thai hypertensive patients from 2014 to 2015.

## Main text

### Methods

A nationwide, cross-sectional survey was conducted from 2014 to 2015. The survey aimed to determine outcomes among patients with hypertension visiting clinics in Bangkok supported by the Thailand National Health Security Office’s (NHSO) program, private hospitals and public hospitals of the Ministry of Public Health (MoPH) in Thailand.

This study was reviewed and approved by the Royal Thai Army Medical Department and local institutional review boards of the participating hospitals. For selecting national representative samples of patients with hypertension in Thailand, a stratified two-stage cluster sampling method proportional to the size was conducted. For Bangkok, the targeted hospitals included all hospitals and clinics under the NHSO. However, all university hospitals were excluded.

Hypertensive patients aged ≥ 18 years receiving medical treatment in a hospital, drawn from those sampling methods, during the previous 1 year were included. Any patient who had participated in a clinical trial was excluded. The number of participants totaled 65,667. The number of hospitals under the MoPH totaled 833 hospitals, including 717 community hospitals, 83 general hospitals and 33 regional hospitals. Hypertensive patients were selected by sampling and registered at each hospital. A standardized case report form was used to collect data from medical records of hypertensive treatment and was sent to the central data management unit. The data, including status of hypertensive complications and results of laboratory tests, were retrieved from patient’s medical records.

Hypertension was defined by JNC’s eight hypertension guidelines as high BP (SBP ≥ 140 mmHg or DBP ≥ 90 mmHg) and/or use of antihypertensive medicine [4]. Uncontrolled hypertension was defined by JNC’s eight hypertension guidelines. The BP target is SBP < 140 mmHg or DBP < 90 mmHg among adults younger than 60 years and a BP goal of SBP < 150 mmHg and DBP < 90 mmHg in the general population aged ≥ 60 years [4]. BMI was calculated as body weight in kilograms (kg) divided by height in meters squared [weight (kg)/height (m)^2^]. Waist circumferences were measure at the level of the umbilicus [5].

Data were coded, entered and analyzed using IBM SPSS Statistics for Windows, Version 23.0. Categorical data were presented as number and percentage, while continuous data were presented as mean and standard deviation (SD). Prevalence was analyzed using descriptive statistics and reported as percentage and 95% confidence interval. The Chi-square test was used to compare categorical data, while continuous data were compared using the *t*-test. The magnitude of association was presented as crude odds ratios (ORs) with 95% confidence interval. The final model with multivariate logistic regression analysis was created. A *p*-value < 0.05 was considered statistically significant.

### Results

A total of 65,667 Thai patients with hypertension were enrolled in this study. The greater proportion of participants, 40,834 (62.2%), were females (Table 1). The average age of participants was 63.9 ± 11.7 years, while the average duration of hypertension was 6.5 ± 4.1 years. The average SBP was 133.6 ± 15.2 mmHg while the average DBP was 75.9 ± 10.6 mmHg. Overall prevalence of uncontrolled hypertension in the study was 24.6% (95% CI 24.2–24.9). Among males and females, uncontrolled hypertension was 25.6% (95% CI 25.1–26.2) and 23.9% (95% CI 23.5–24.3), respectively. Univariate and multivariate analysis were performed to determine factors associated with uncontrolled hypertension (Tables 2 and 3). The final model was adjusted for sex, age, regions, hospital levels, smoking, diabetes mellitus (DM) comorbidity, duration of hypertension, BMI, LDL level and the number of antihypertensive medications used. After adjusting, the factors associated with uncontrolled hypertension comprised being male, regions, hospital levels, DM comorbidity, higher BMI, higher LDL level and higher number of antihypertensive medications used; however, higher age was a protective factor for uncontrolled hypertension.

**Table 1:** Demographic characteristics of the enrolled Thai adults with hypertension

Variables	n (%)
Gender	
Female	40,834 (62.2)
Male	24,833 (37.8)
Age (years) (mean ± SD)	63.9 ± 11.7
20–29	98 (0.1)
30–39	1077 (1.6)
40–49	6383 (9.7)
50–59	16,276 (24.8)
60–69	19,966 (30.4)
70–79	15,511 (23.6)
80–89	5832 (8.9)
≥ 90	456 (0.7)
Region, n (%)	
Northeast	14,864 (22.6)
Northern	18,032 (27.5)
Central	23,104 (35.2)
Southern	9667 (14.7)
Hospital level	
First level	45,541 (69.4)
Middle level	15,030 (22.9)
Standard/Advance level	5096 (7.8)
Healthcare coverage	
Universal coverage scheme	48,747 (74.2)
Government officer	13,181 (20.1)
Social security scheme	2931 (4.5)
Others	808 (1.2)
Occupations	
Unemployed/retired	22,438 (34.2)
Agriculturist	3346 (5.1)
Private business	22,649 (34.5)
Officer	3820 (5.8)
Others	13,414 (20.4)
Comorbidity	
Dyslipidemia	41,171 (62.7)
Diabetes mellitus	8628 (13.1)
Gout	5083 (7.7)
Smoking	
Never	50,733 (82.6)
Ex-smoker	7624 (12.4)
Current smoker	3035 (4.9)
Duration of hypertension (years) (mean ± SD)	6.5 ± 4.1
Blood pressure (mmHg)	
SBP (mean ± SD)	132.6 ± 15.2
DBP (mean ± SD)	75.9 ± 10.6
Waist circumference (cm)	86.3 ± 10.8
BMI (kg/m^2^) (mean ± SD)	24.9 ± 4.7
< 25	34,137 (54.1)
25–29.99	20,797 (33)
30–34.99	6386 (10.1)
≥ 35	1751 (2.8)
Biochemical measurements (mean ± SD)	
Serum uric acid (mg/dL)	6.1 ± 1.8
TC (mg/dL)	193.7 ± 44.7
TG (mg/dL)	150.9 ± 89.2
HDL (mg/dL)	50.7 ± 15.6
LDL (mg/dL)	115.1 ± 36.8
Estimated GFR (mL/min/1.73 m^2^)	71 ± 23.6
≥ 90	13,678 (23.7)
60–89	25,263 (43.8)
30–59	16,418 (28.4)
15–29	1649 (2.9)
< 15	731 (1.3)
Number of antihypertensive medications	
0	1110 (1.7)
1	26,153 (39.8)
2	27,175 (41.4)
≥ 3	11,229 (17.1)

*SD* standard deviation, *mmHg* millimeter of mercury, *SBP* systolic blood pressure, *DBP* diastolic blood pressure, *cm* centimeter, *FPG* fasting plasma glucose, *TC* total cholesterol, *TG* triglyceride, *HDL*, high-density lipoprotein cholesterol, *LDL* low-density lipoprotein cholesterol, *BMI* body mass index, *eGFR* estimated glomerulus filtration rate

**Table 2:** Univariate analysis for factors associated with uncontrolled hypertension among Thai adults with hypertension

Variables	Normotension	Uncontrolled HT	Crude	95% CI	*p*-value
	n (%)	n (%)	Odds ratio		
Gender					
Female	31,077 (76.1)	9757 (23.9)	1		
Male	18,468 (74.4)	6365 (25.6)	1.10	(1.06–1.14)	< 0.001
Age (years)	64.9 ± 11.4	60.7 ± 12.2	0.97	(0.97–0.97)	< 0.001
20–29	48 (49.0)	50 (51.0)	1		
30–39	625 (58.0)	452 (42.0)	0.69	(0.46–1.05)	0.084
40–49	4064 (63.7)	2319 (36.3)	0.55	(0.37–0.82)	0.003
50–59	10,811 (66.4)	5465 (33.6)	0.49	(0.33–0.72)	< 0.001
60–69	16,251 (81.4)	3715 (18.6)	0.22	(0.15–0.33)	< 0.001
70–79	12,613 (81.3)	2898 (18.7)	0.22	(0.15–0.33)	< 0.001
80–89	4697 (80.5)	1135 (19.5)	0.23	(0.16–0.35)	< 0.001
≥ 90	384 (84.2)	72 (15.8)	0.18	(0.11–0.29)	< 0.001
Region					
Northeast	11,813 (79.5)	3051 (20.5)	1		
Northern	13,717 (76.1)	4315 (23.9)	1.22	(1.16–1.28)	< 0.001
Central	17,101 (74.0)	6003 (26.0)	1.36	(1.29–1.43)	< 0.001
Southern	6914 (71.5)	2753 (28.5)	1.54	(1.45–1.64)	< 0.001
Hospital level					
First level	35,160 (77.2)	10,381 (22.8)	1		
Middle level	10,668 (71.0)	4362 (29.0)	1.39	(1.33–1.44)	< 0.001
Standard/advance level	3717 (72.9)	1379 (27.1)	1.26	(1.18–1.34)	< 0.001
Healthcare coverage					
Universal coverage scheme	36,881 (75.7)	11,866 (24.3)	1		
Government officer	10,193 (77.3)	2988 (22.7)	0.91	(0.87–0.95)	< 0.001
Social security scheme	1903 (64.9)	1028 (35.1)	1.68	(1.55–1.82)	< 0.001
Others	568 (70.3)	240 (29.7)	1.31	(1.13–1.53)	< 0.001
Occupations					
Unemployed/retired	17,809 (79.4)	4629 (20.6)	1		
Agriculturist	2387 (71.3)	959 (28.7)	1.55	(1.43–1.68)	< 0.001
Private business	17,266 (76.2)	5383 (23.8)	1.20	(1.15–1.25)	< 0.001
Officer	2706 (70.8)	1114 (29.2)	1.58	(1.47–1.71)	< 0.001
Others	9377 (69.9)	4037 (30.1)	1.66	(1.58–1.74)	< 0.001
Dyslipidemia					
No	18,345 (74.9)	6151 (25.1)	1		
Yes	31,200 (75.8)	9971 (24.2)	0.95	(0.92–0.99)	0.010
Diabetes mellitus					
No	43,311 (75.9)	13,728 (24.1)	1		
Yes	6234 (72.3)	2394 (27.7)	1.21	(1.15–1.28)	< 0.001
Gout					
No	45,755 (75.5)	14,829 (24.5)	1		
Yes	3790 (74.6)	1293 (25.4)	1.05	(0.99–1.12)	0.126
Smoking					
Never	38,552 (76.0)	12,181 (24.0)	1		
Ex-smoker	5801 (76.1)	1823 (23.9)	0.99	(0.94–1.05)	0.851
Current smoker	2202 (72.6)	833 (27.4)	1.20	(1.10–1.30)	< 0.001
Duration of hypertension (years)	6.6 ± 4.1	6.4 ± 4.0	0.99	(0.98–0.99)	< 0.001
Waist circumference (cm)	85.8 ± 10.6	87.8 ± 11.1	1.02	(1.01–1.02)	< 0.001
BMI (kg/m^2^)	24.6 ± 4.5	25.9 ± 4.9	1.06	(1.06–1.06)	< 0.001
< 25	27,156 (79.6)	6981 (20.4)	1		
25–29.99	15,227 (73.2)	5570 (26.8)	1.42	(1.37–1.48)	< 0.001
30–34.99	4309 (67.5)	2077 (32.5)	1.88	(1.77–1.99)	< 0.001
≥ 35	1048 (59.9)	703 (40.1)	2.61	(2.36–2.88)	< 0.001
Biochemical measurements					
Serum uric acid (mg/dL)	6.1 ± 1.8	6.2 ± 1.9	1.04	(1.03–1.06)	< 0.001
TC (mg/dL)	192.3 ± 43.1	198.1 ± 49.0	1.01	(1.00–1.01)	< 0.001
TG (mg/dL)	148.8 ± 87.3	157.5 ± 94.6	1.01	(1.00–1.01)	< 0.001
HDL (mg/dL)	50.7 ± 15.8	50.8 ± 15.2	1.01	(1.00–1.01)	0.467
LDL (mg/dL)	114.0 ± 36.4	118.5 ± 38.1	1.01	(1.00–1.01)	< 0.001
Estimated GFR (mL/min/1.73 m^2^)	70.2 ± 23.2	73.7 ± 24.7	1.01	(1.00–1.01)	< 0.001
≥ 90	9732 (71.2)	3946 (28.8)	1		
60–89	19,314 (76.5)	5949 (23.5)	0.76	(0.73–0.80)	< 0.001
30–59	12,974 (79.0)	3444 (21.0)	0.66	(0.62–0.69)	< 0.001
15–29	1274 (77.3)	375 (22.7)	0.73	(0.64–0.82)	< 0.001
< 15	511 (69.9)	220 (30.1)	1.06	(0.90–1.25)	0.469
Number of antihypertensive medications					
0	930 (83.8)	180 (16.2)	1		
1	20,717 (79.2)	5436 (20.8)	1.36	(1.15–1.60)	< 0.001
2	20,148 (74.1)	7027 (25.9)	1.80	(1.53–2.12)	< 0.001
≥ 3	7750 (69.0)	3479 (31.0)	2.32	(1.97–2.73)	< 0.001
Drug classes (monotherapy)					
ARB	1953 (76.6)	595 (23.4)	1		
ACEI	5520 (77.1)	1644 (22.9)	0.98	(0.88–1.09)	0.678
Anti-adrenergic	2060 (78.4)	566 (21.6)	0.90	(0.79–1.03)	0.121
Diuretics	2697 (80.9)	635 (19.1)	0.77	(0.68–0.88)	< 0.001
CCBs	8301 (81.2)	1921 (18.8)	0.76	(0.68–0.84)	< 0.001

*SD* standard deviation, *mmHg* millimeter of mercury, *SBP* systolic blood pressure, *DBP* diastolic blood pressure, *cm* centimeter, *FPG* fasting plasma glucose, *TC* total cholesterol, *TG* triglyceride, *HDL*, high-density lipoprotein cholesterol, *LDL* low-density lipoprotein cholesterol, *BMI* body mass index, *eGFR* estimated glomerulus filtration rate, *ARB* angiotensin receptor blockers, *ACEI* angiotensin converting enzyme inhibitors; anti-adrenergic; alpha and beta blockers, *CCBs*, calcium channel blockers

**Table 3:** Multivariate analysis for factors associated with uncontrolled hypertension among Thai adults with hypertension

Variables	Adjusted odds ratio	95% CI	*p*-value
Gender			
Female	1		
Male	1.16	(1.10–1.20)	< 0.001
Age (years)	0.97	(0.97–0.97)	< 0.001
Region			
Northeast	1		
Northern	1.21	(1.14–1.29)	< 0.001
Central	1.20	(1.13–1.28)	< 0.001
Southern	1.48	(1.38–1.59)	< 0.001
Hospital level			
First level	1		
Middle level	1.23	(1.16–1.30)	< 0.001
Standard/advance level	1.12	(1.03–1.22)	0.009
Diabetes mellitus			
No	1		
Yes	1.16	(1.08–1.23)	< 0.001
Smoking			
Never	1		
Ex-smoker	1.04	(0.96–1.11)	0.334
Current smoker	1.08	(0.97–1.19)	0.155
Duration of hypertension (years)	1.00	(0.99–1.01)	0.120
BMI (kg/m^2^)			
< 25	1		
25–29.99	1.19	(1.13–1.24)	< 0.001
30–34.99	1.42	(1.32–1.52)	< 0.001
≥ 35	1.70	(1.51–1.92)	< 0.001
LDL (mg/dL)			
< 100	1		
> 100	1.17	(1.11–1.22)	< 0.001
Number of antihypertensive medications			
0	1		
1	1.39	(1.13–1.70)	0.002
2	1.80	(1.47–2.20)	< 0.001
≥ 3	2.32	(1.89–2.86)	< 0.001

*LDL* low-density lipoprotein cholesterol, *BMI* body mass index

Adjusted for gender, age, regions, hospital levels, diabetes comorbidity, smoking status, duration of hypertension, body mass index, LDL level and number of antihypertensive medications

### Discussion

These results revealed important implications to the Thai public health system because hypertension is a major risk factor for cerebrovascular events and cardiovascular diseases. A secondary intervention is necessary to reduce any complications. This study comprised the largest research in Southeast Asia, focusing on uncontrolled hypertension. The present nationwide survey from 2014 to 2015 showing the prevalence of uncontrolled hypertension among Thai adults with hypertension was 24.6%. The prevalence of uncontrolled hypertension in the current study was lower than that previously reported for US and China [6, 7]. However, in Thailand one related study in 2009 reported the prevalence of uncontrolled hypertension was 75.6% [2]. Thus, in the present study, the prevalence of uncontrolled hypertension was lower than that in the 2009 survey. This situation could be explained by several points. Firstly, the related study [2] was conducted by a national health examination in a field survey while the present study was conducted among hypertensive patients receiving medical treatment in a hospital. In 2012, Thai guidelines on treating hypertension were released to direct any physician including general practitioners and specialists. Therefore, Thai patients with hypertension have been receiving appropriate medical treatment. Consequently, patients with hypertension, who follow-up with their physicians, have obtained more proper medical care. In the present study, patients with hypertension obtaining medical treatment in hospitals under the MoPH all over Thailand were enrolled. However, those with high BP treated at primary care units or university hospitals were not included in the current study; thus, the prevalence of uncontrolled hypertension may be underestimated.

The present study showed that patients with hypertension at higher ages tended to be at lower risk for uncontrolled hypertension, similar to other studies [7, 8]. Hypertensive complications and end organ damage will not occur in the early period of hypertension. Thus, younger hypertensive patients lack awareness of complications; moreover, working age people may not follow up their appointment with medical doctors resulting from the available time mismatch between working age patients and healthcare services [9]. However, some studies have shown that the prevalence of uncontrolled hypertension among elderly hypertensive patients was more likely to be higher [10–12]. Similarly, some related studies conducted in the US and China revealed that males had a higher risk for uncontrolled hypertension than females [10, 13–15] due to biological factors [16] including hormonal effects on increased BP. These related studies have shown that females have lower pro-renin and renin levels than males, causing BP among males to be higher than females [16, 17]. Nevertheless, another study reported hypertension could be better controlled among males [18].

Our findings showed that the prevalence of uncontrolled hypertension differed by region. In the northeast area, the prevalence of uncontrolled hypertension was significantly lower than that in other regions. The finding could be explained by behavioral and local cultural factors. Patients in this area enjoyed consuming vegetable and fruits, which may have decreased body adiposity levels, consequently reducing BP [19–21]. Nevertheless, after adjusting, both residing in northeast Thailand and BMI were associated with controlled BP. Another explanation is that northeast Thailand is mainly an agricultural area. As a result, the patients with hypertension in this area are agriculturists; moreover, traditional dancing and playing of northeastern Thai games creates more physical activities. More physical activities can decrease both systolic and diastolic BP including decreased risks for high BP [22, 23]. Our study has shown that hypertensive patients receiving medical treatment at middle level hospitals and standard/advanced level hospitals tended to indicate a higher prevalence of uncontrolled hypertension compared with those at first level hospitals. Similarly, one related study in Kenya illustrated that hypertensive patients in provincial hospitals could control their BP more effectively than those in national hospitals [24]. Patients with uncontrolled hypertension in first level hospitals may be referred to high potential hospitals for proper medical management. Consequently, high level hospitals are permeated with hypertensive patients with poorly controlled BP.

The study reported hypertensive patients with diabetes comorbidity were associated with BP control. This result was similar to that of related studies in Europe [25, 26], showing that diabetes was associated with a high risk of poor BP control. This effect may have resulted from insulin resistance and endothelial dysfunction; thus, increasing BP [27–29]. Several studies reported similar results with this study revealing a relationship between BMI and controlled BP [30–33]. The study found that patients with hypertension presenting BMI more than 25 kg/m^2^ tended to be higher at risk of uncontrolled BP. Notably, patients with BMI > 35 kg/m^2^ were 1.7 times at higher risk when compared with those with BMI < 25 kg/m^2^. This finding could be attributed among most patients with higher BMI defined as obesity. Most patients with obesity have high adiposity levels. The adipocytes are substrate for producing leptin, resulting in increased leptin level, leading to heightened sympathetic nerve activity, contributing to elevated BP [34, 35]. The present study reported that increasing LDL level was associated with uncontrolled hypertension. Our findings agree with related studies [26, 36, 37] supporting the relationship between the cholesterol level and increased BP. Evidence supports the results that LDL cholesterol elevation leads to increased stability of mRNA for AT1 receptors regarding angiotensin II. Moreover, LDL density at vascular level is proportional to cholesterolemia, contributing to vasoconstriction and pressure in response to angiotensin II infusion [38].

Our data showed a relationship between increasing amounts of antihypertensive medications used and uncontrolled BP, firstly, illustrating that other studies reported similar results, followed by various factors [39, 40]. Patients experiencing difficulty controlling BP are likely to be treated with multiple medications. Therefore, this result might be a consequence of uncontrolled hypertension. However, we found that some patients with hypertension without using antihypertensive medication could control their BP.

The strength of this study includes its nationwide scope for uncontrolled hypertension, the largest sample size in Southeast Asia. Thus, the findings of the study could be generalized and applied in similar hypertension populations. One implication of the study is to reduce the prevalence of uncontrolled hypertension, resulting from improving hypertension management. Moreover, hypertensive patients should control their modifiable risk factors. Healthcare services access of hypertensive patients especially at working age should be adjusted for appropriate situations. Above all, MoPH’s managers and physicians should provide further preventative strategies to attenuate cardiovascular risk factors.

## Limitations

The study employed a cross-sectional design, and as such, the results could show only factors associated with uncontrolled hypertension. The data presented in the present study were obtained in the 2014 to 2015 Thailand DM/HT of the NHSO from MedResNet central data management, so we were aware of missing data from this observational study. However, this represents a relatively large sample size and some data were missing as from the nationwide observational study, so associations between factors and outcomes could be presented.

## Data Availability

The datasets generated and/or analyzed during the current study are available at http://www.damus.in.th after the permission of the Thailand DM/HT study of the Medical Research Network of the Consortium of Thai Medical Schools (MedResNet).

## Abbreviations

DM: diabetes mellitus
HT: hypertension
SBP: systolic blood pressure
DBP: diastolic blood pressure
CI: confidence interval
BMI: body mass index
MoPH: Ministry of Public Health
NHSO: National Health Security Office
SD: standard deviation
GFR_EPI: glomerular infiltration rate calculated by epidemiology collaboration formula
Hb: hemoglobin
Hct: hematocrit
LDL: low density lipoprotein cholesterol
HDL: high density lipoprotein cholesterol
TG: triglyceride
TC: total cholesterol
